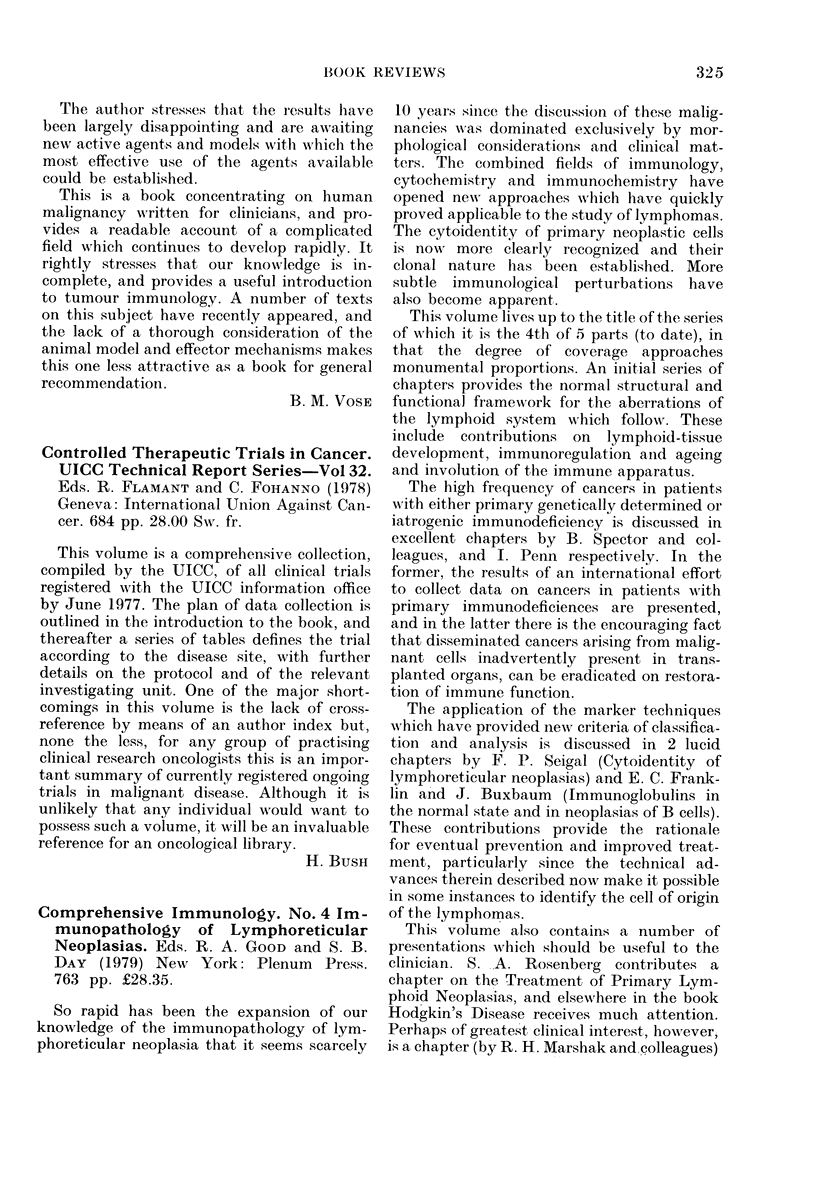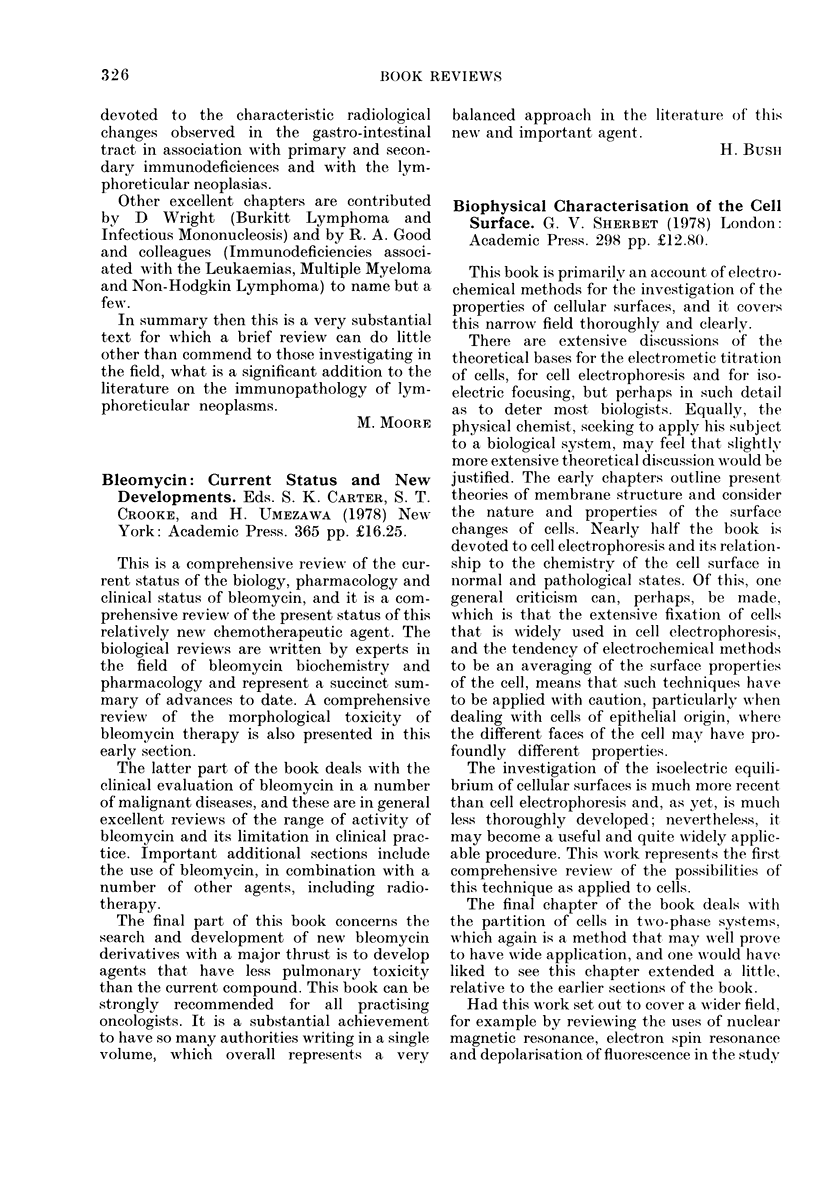# Comprehensive Immunology. No. 4 Immunopathology of Lymphoreticular Neoplasias

**Published:** 1979-08

**Authors:** M. Moore


**Comprehensive Immunology. No. 4 Immunopathology of Lymphoreticular Neoplasias.** Eds. R. A. Good and S. B. Day (1979) New York: Plenum Press. 763 pp. £28.35.

So rapid has been the expansion of our knowledge of the immunopathology of lymphoreticular neoplasia that it seems scarcely 10 years since the discussion of these malignancies was dominated exclusively by morphological considerations and clinical matters. The combined fields of immunology, cytochemistry and immunochemistry have opened new approaches which have quickly proved applicable to the study of lymphomas. The cytoidentity of primary neoplastic cells is now more clearly recognized and their clonal nature has been established. More subtle immunological perturbations have also become apparent.

This volume lives up to the title of the series of which it is the 4th of 5 parts (to date), in that the degree of coverage approaches monumental proportions. An initial series of chapters provides the normal structural and functional framework for the aberrations of the lymphoid system which follow. These include contributions on lymphoid-tissue development, immunoregulation and ageing and involution of the immune apparatus.

The high frequency of cancers in patients with either primary genetically determined or iatrogenic immunodeficiency is discussed in excellent chapters by B. Spector and colleagues, and I. Penn respectively. In the former, the results of an international effort to collect data on cancers in patients with primary immunodeficiences are presented, and in the latter there is the encouraging fact that disseminated cancers arising from malignant cells inadvertently present in transplanted organs, can be eradicated on restoration of immune function.

The application of the marker techniques which have provided new criteria of classification and analysis is discussed in 2 lucid chapters by F. P. Seigal (Cytoidentity of lymphoreticular neoplasias) and E. C. Franklin and J. Buxbaum (Immunoglobulins in the normal state and in neoplasias of B cells). These contributions provide the rationale for eventual prevention and improved treatment, particularly since the technical advances therein described now make it possible in some instances to identify the cell of origin of the lymphomas.

This volume also contains a number of presentations which should be useful to the clinician. S. A. Rosenberg contributes a chapter on the Treatment of Primary Lymphoid Neoplasias, and elsewhere in the book Hodgkin's Disease receives much attention. Perhaps of greatest clinical interest, however, is a chapter (by R. H. Marshak and colleagues)

devoted to the characteristic radiological changes observed in the gastro-intestinal tract in association with primary and secondary immunodeficiences and with the lymphoreticular neoplasias.

Other excellent chapters are contributed by D Wright (Burkitt Lymphoma and Infectious Mononucleosis) and by R. A. Good and colleagues (Immunodeficiencies associated with the Leukaemias, Multiple Myeloma and Non-Hodgkin Lymphoma) to name but a few.

In summary then this is a very substantial text for which a brief review can do little other than commend to those investigating in the field, what is a significant addition to the literature on the immunopathology of lymphoreticular neoplasms.

M. Moore